# Supplementary figures and images for: Discrimination of Bacterial Community Structures among Healthy, Gingivitis, and Periodontitis Statuses through Integrated Metatranscriptomic and Network Analyses
Source: mSystems. 2021 Oct 26;6(6):e00886-21. doi: 10.1128/mSystems.00886-21 (PMC8547322; doi:10.1128/mSystems.00886-21)

(A)

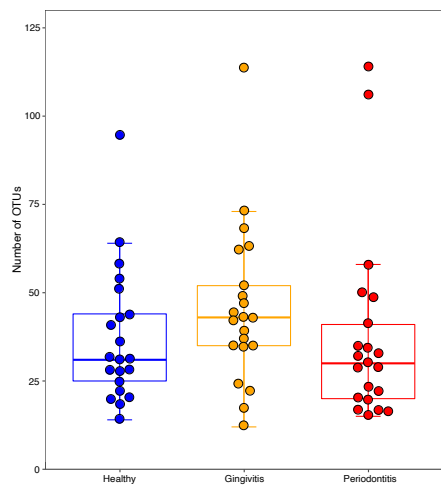

(B)

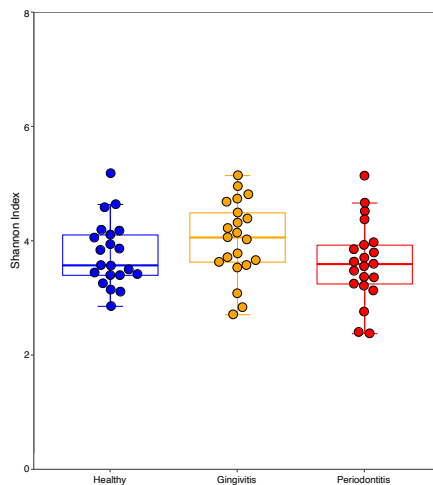

(C)

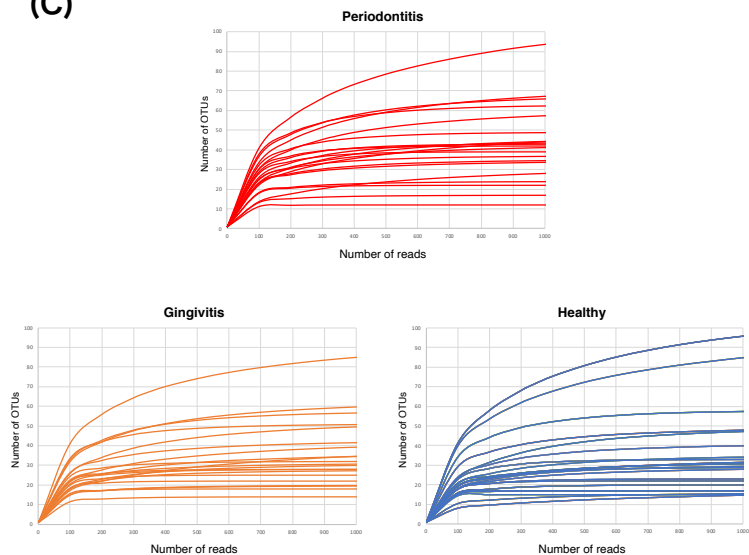

Supplement: FIG S1 [file msystems.00886-21-sf001.pdf]

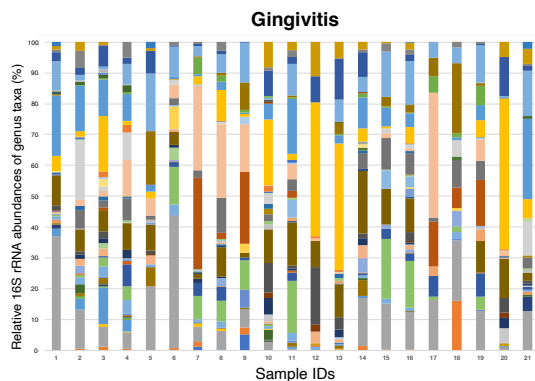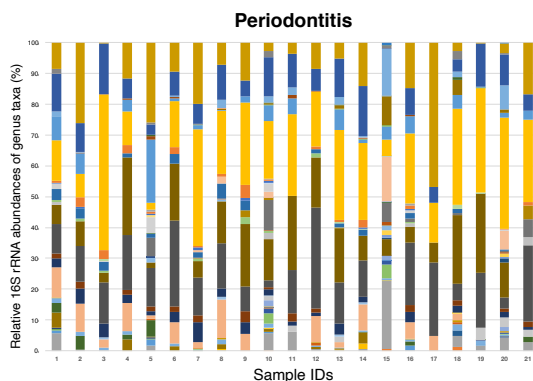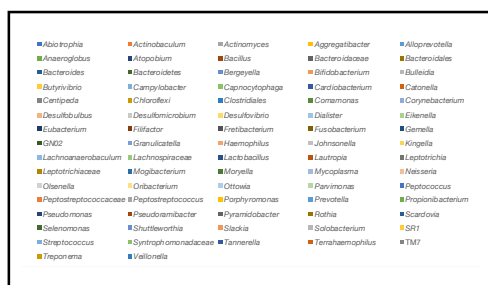

Supplement: FIG S2 [file msystems.00886-21-sf002.pdf]

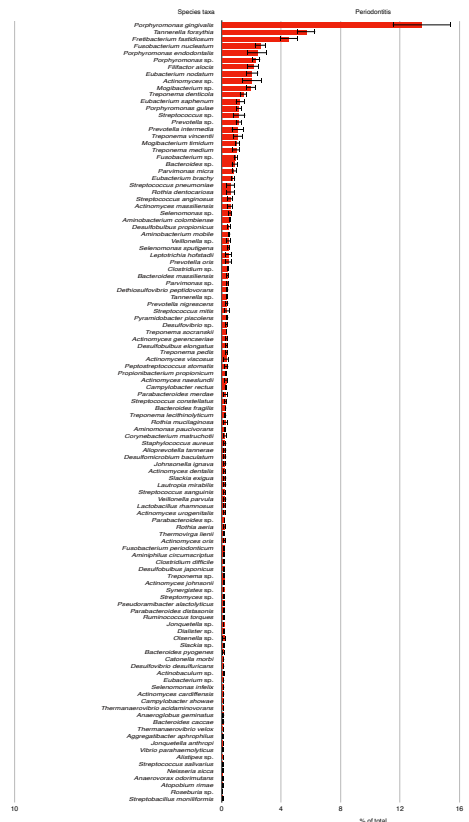

Supplement: FIG S3 [file msystems.00886-21-sf003.pdf]

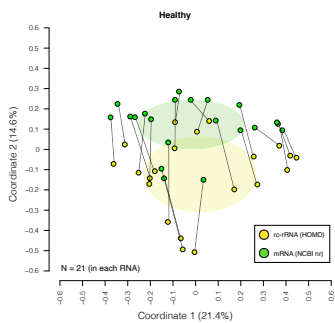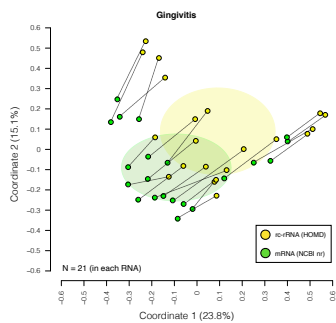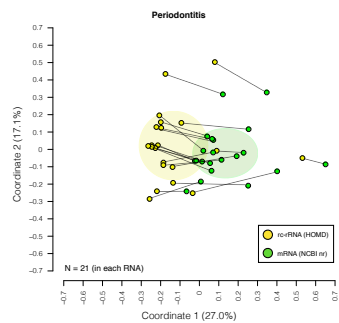

Supplement: FIG S4 [file msystems.00886-21-sf004.pdf]

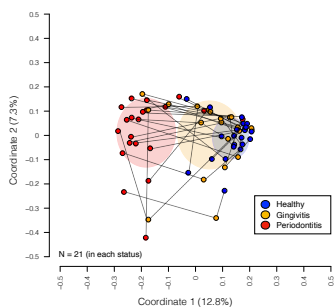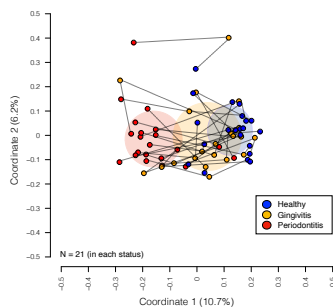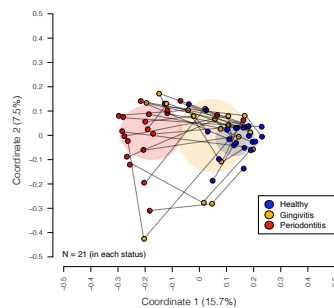

Supplement: FIG S5 [file msystems.00886-21-sf005.pdf]
