# Supplementary material for: Discrimination of Bacterial Community Structures among Healthy, Gingivitis, and Periodontitis Statuses through Integrated Metatranscriptomic and Network Analyses
Source: mSystems. 2021 Oct 26;6(6):e00886-21. doi: 10.1128/mSystems.00886-21 (PMC8547322; doi:10.1128/mSystems.00886-21)
Supplement: TEXT S1 [file msystems.00886-21-s0001.docx]

**Supplementary Results**

**Comparison of functional profiles among microbiomes**

Based on analysis using the National Center for Biotechnology Information non-redundant (NCBI nr) protein database, mRNA clusters were first assigned descriptions of the bacterial genes. In total, 37,814 bacterial genes were identified; 24,742, 22,513, and 15,423 genes were identified in H, G, and P sites, respectively (Table S1E). Furthermore, 7,573 genes were common among all three sites; of these, most were genes encoding membrane proteins (H: 1.92% ± 0.55%, G: 1.78% ± 0.47%, and P: 1.44% ± 0.44%). Assessments by PCoA (Figure S5A) and PERMANOVA revealed dissimilar mRNA profiles among the three periodontal statuses (F = 2.36 and *p* = 1.00E-4).

**Functional profiles of putative virulence factors**

n total, 2,989, 2,909, and 2,208 putative virulence genes based on mRNA clusters in the Virulence Factors of Pathogenic Bacteria database (VFDB) were detected in H, G, and P sites, respectively (Table S1F); 1,295 genes were common among all three sites. Elongation factor Tu related genes exhibited high prevalence in three sites. Especially, the translation elongation factor Tu gene was most prevalent among the three sites (H: 4.32% ± 1.16%, G: 4.65% ± 2.04%, and P: 4.07% ± 1.57%). Moreover, alkyl hydroperoxide reductase, superoxide dismutase, and glyceraldehyde 3-phosphate dehydrogenase were considerably prevalent in all periodontal statuses. The profiles of putative virulence factors, based on VFDB analyses, were dissimilar among the three periodontal statuses, as shown in dendrograms and PCoA plots (Figure S5B), as well as PERMANOVA assessment (F = 2.637 and *p* = 1.00E-4). The mRNA abundances of 16 putative virulence factor genes (e.g., putative alcohol-acetaldehyde dehydrogenase, and SPI-3 pathogenicity island encoded protein) significantly differed among the three sites (*p* < 0.005); most significant differences in putative virulence factor expression were detected between H and P sites.

MvirDB-focused analysis revealed the presence of 1,835, 1,824 and 1,463 virulence genes based on mRNA clusters in H, G, and P sites, respectively (Table S1G); 1,082 genes were common among all three periodontal statuses. The translation initiation factor IF-1 gene was most prevalent among the three periodontal statuses (H: 7.20% ± 4.05%, G: 6.77% ± 4.43%, and P: 3.37% ± 2.03%); Other genes related to ribosomal protein also exhibited considerable prevalence in three periodontal statuses. Assessments by PCoA (Figure S5C) and PERMANOVA (F = 4.260 and *p* = 1.00E-4) showed dissimilar mRNA profiles among the three periodontal statuses. Thirty-six putative virulence factor genes (e.g., ferredoxin oxidoreductase A-like protein and 4-hydroxybutyrate coenzyme A transferase) exhibited significantly different expression among the three periodontal statuses (*p* < 0.005). Similar to the VFDB-focused analysis, most differences in putative virulence factor expression were found between H and P sites.
